# Antimicrobial proteins regulating neuroinflammation

**DOI:** 10.1080/07853890.2025.2610072

**Published:** 2026-01-05

**Authors:** Anup Bhusal, Won-Ha Lee, Kyoungho Suk

**Affiliations:** ^a^ Department of Pharmacology, School of Medicine, Kyungpook National University, Daegu, Republic of Korea; ^b^ Board of Governors Regenerative Medicine Institute, Cedars-Sinai Medical Center, Los Angeles, CA, USA; ^c^ BK21 Plus KNU Biomedical Convergence Program, Department of Biomedical Science, School of Medicine, Kyungpook National University, Daegu, Republic of Korea; ^d^ Brain Science and Engineering Institute, Kyungpook National University, Daegu, Republic of Korea; ^e^ School of Life Sciences, BK21 plus KNU Creative BioResearch Group, Kyungpook National University, Daegu, Republic of Korea

**Keywords:** Antimicrobial peptides/proteins, glia, defense, neuroinflammation, neurological disorders

## Abstract

**Background:**

Antimicrobial peptides/proteins (AMPs), also termed host defense peptides, are well-known effector molecules in innate immunity across all organisms. Recent research has revealed that AMPs possess multifunctional properties beyond their antimicrobial activity, including roles in neuroinflammation. The expression of AMPs has been detected within the central nervous system (CNS) at a basal level and is generally upregulated in various neurological disorders. Owing to its expression and induction in the CNS, there has been growing interest in investigating the relationships among AMPs, neuroinflammation, and various neurological disorders.

**Methods:**

To ensure a comprehensive overview, relevant articles were identified through an extensive PubMed search for this review. Here, we discuss recent literature and advances in understanding AMPs in the CNS at both molecular and functional levels. Additionally, the potential use of these proteins in targeting neuroinflammatory disorders is examined.

**Conclusions:**

Overall, this review provides insight into the complex interplay between AMPs and neuroinflammation and highlights the need for further research in this field.

## Introduction

The innate immune system is the initial line of defense against infections and is characterized by its rapid response and broad-spectrum action. Antimicrobial peptides and proteins (AMPs) are a diverse class of naturally occurring molecules that play a crucial role in the innate immune response [1]. Since their discovery, several thousands of AMPs have been identified and isolated across various natural sources like microorganisms, plants, insects, crustaceans, animals, humans, etc [2]. The diversity of AMPs discovered is so great that it is difficult to categorize; AMPs generally include cationic antimicrobial peptides, S100 family proteins, peptidoglycan-recognition proteins, calcium-dependent lectins and iron metabolism-related proteins [3].

**Figure 1.**
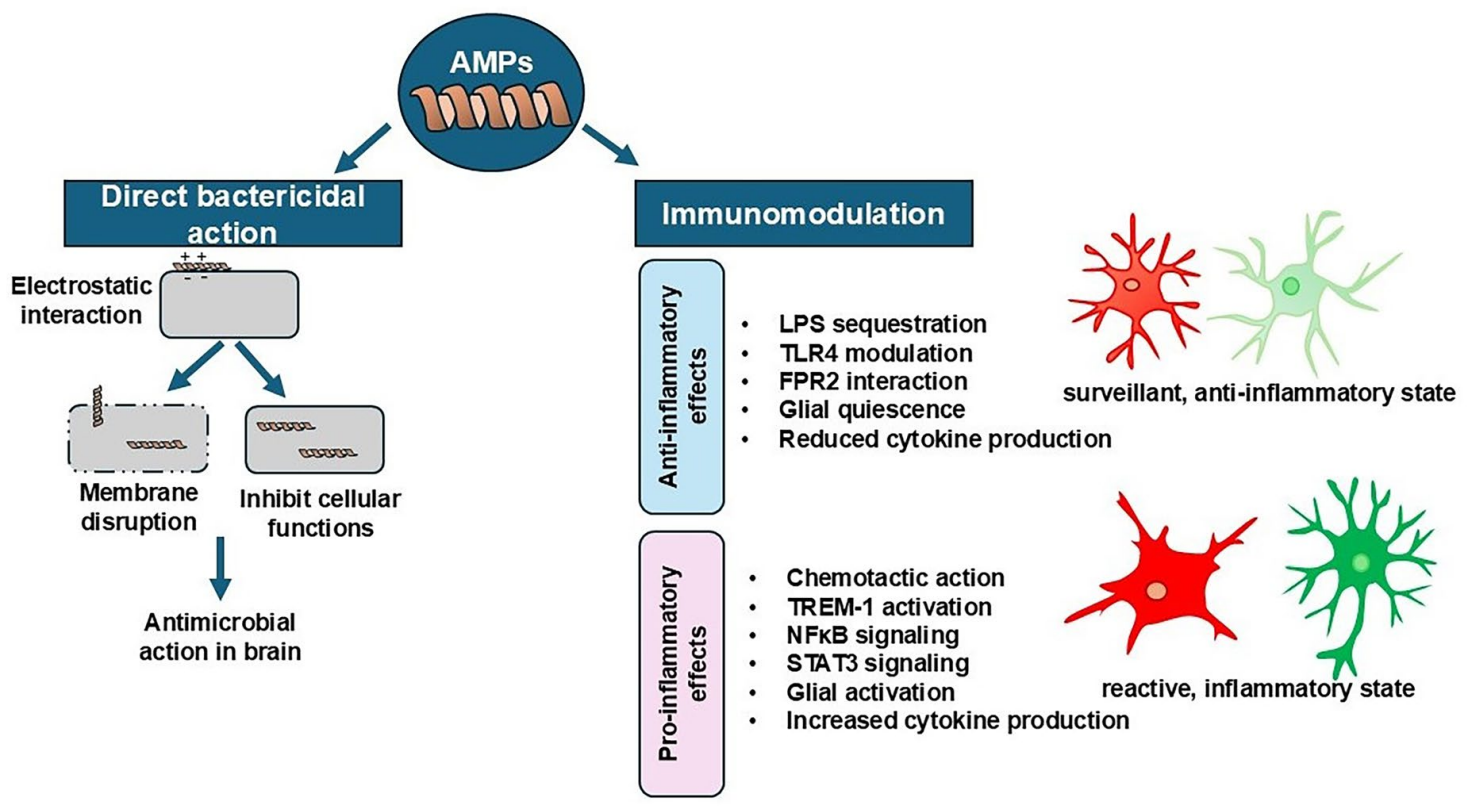
Role of AMPs in host defense and immune regulation in brain. AMPs may contribute to host defense *via* two major mechanisms: (1) Direct antimicrobial action by disrupting bacterial membranes or interfering with intracellular targets, leading to microbial death; and (2) immunomodulation, involving both anti-inflammatory and pro-inflammatory effects on host immune cells. AMPs can promote an anti-inflammatory or pro-inflammatory states—supporting immune homeostasis and resolution of inflammation or enhance pro-inflammatory responses. Representative glial cells are depicted in as representative immune effectors. In the homeostatic state, microglia appear light red with a highly branched morphology, indicative of their surveillant, anti-inflammatory role. Upon activation, they become reactive, adopting an amoeboid shape with fewer or no processes and a darker red colour, reflecting increased inflammatory reactivity. Similarly, astrocytes in the homeostatic condition are depicted as light green with fewer, thinner branches, supporting normal CNS function. In response to pro-inflammatory signals, astrocytes become reactive, showing a brighter green colour and increased branching, representing their shift towards a more active, possibly neuroprotective or pro-inflammatory role depending on the context. Abbreviations: LPS- Lipopolysaccharide; TLR-Toll-like receptor; FPR- Formyl peptide receptor; TREM- Triggering receptor expressed on myeloid cells; NFκB-Nuclear factor kappa B; Stat-Signal transducer and activator of transcription.

AMPs are produced by various cells and tissues, including immune cells, epithelial cells and mucosal surfaces, and their significance stems from their potent antimicrobial activity and ability to modulate immune responses. They carry out their function by interacting with the membrane of the microbial cell, compromising its integrity and causing cell lysis or death [4]. Furthermore, AMPs influence immune cells, leading them to release pro-inflammatory cytokines and chemokines while recruiting additional immune cells to the site of infection or injury, thereby further modulating immune responses [4–6]. On the other hand, AMPs also possess anti-inflammatory properties. They can modulate the immune response by directly neutralizing or sequestering pro-inflammatory molecules to prevent excessive inflammation [7,8]. Overall, AMPs play a significant role in initiating and/or resolving inflammation (Figure 1). Their ability to exhibit antimicrobial activity and modulate immune responses makes them important players in the host defense against infections and the regulation of inflammatory processes.

Traditionally, AMPs have been primarily studied for their antimicrobial properties; however, emerging research has shed light on their involvement in various aspects of neuroinflammation and brain health [9]. Several AMPs, such as defensins, cathelicidins and lipocalin-2, are expressed in the CNS glial cells (such as microglia and astrocytes), neurons and endothelial cells of the blood-brain barrier [9]. They hold promise for modulating immune responses, promoting tissue repair and potentially combating infections in the CNS [9,10]. They can influence the activation and function of immune cells in the CNS, such as microglia, by regulating the release of pro-inflammatory cytokines and other inflammatory mediators. This modulation helps regulate the extent and duration of neuroinflammation and may contribute to maintaining the balance between protective and detrimental immune responses in the brain [11–13]. While the exact mechanisms and functions of AMPs in the brain and neuroinflammation are still being explored, AMPs have gained attention as potential therapeutic agents due to their multifunctional properties. Currently available literature provides a comprehensive overview of AMPs, their sources, structures, mechanisms of action and potential applications [2,14–16]. This mini review aims to delve into the diverse roles of AMPs in the nervous system, particularly within the context of neuroinflammation and neurodegenerative diseases, and it outlines the open questions of this rapidly expanding field. Here, we have selected three representative AMPs for detailed description based on their significant role in neuroinflammation. Whether other AMPs also participate in neuroinflammation remains to be investigated further.

## Cathelicidin-related antimicrobial peptide (CRAMP)

Cathelicidins are a family of small, cationic and amphipathic peptides that play crucial roles in both innate and adaptive immunity. These peptides are initially synthesized as precursor proteins and then cleaved to form biologically active mature peptides [17–19]. The human genome contains only one cathelicidin gene, also known as human cationic antimicrobial peptide-18 (*hCAP-18*), cathelicidin antimicrobial peptide (*CAMP*), or leucine leucine-37 (*LL-37*) [20–24]. This peptide, synthesized initially as a propeptide, is cleaved to form cathelin and the C-terminal LL-37, known for its antimicrobial activity [25]. Similarly, the mouse genome has one cathelicidin gene, cathelicidin-related antimicrobial peptide (*Camp*), which is highly homologous to the human gene [26,27] (Table 1). LL-37 and CRAMP are amphipathic α-helical peptides that bind to and disrupt bacterial cell walls, exhibiting antimicrobial activity [28,29].

**Table 1.** Human and mouse cathelicidin genes, precursors and mature peptide sequences.

Species	Gene Name	Propeptide (precursor)Name	Mature Peptide Name	Mature Peptide Sequence (N-C)	References
Human	CAMP	hCAP-18	LL-37	LLGDFFRKSKEKIGKEFKRIVQRIKDFLRNLVPRTES	[105,106]
Mouse	Camp	Prepro-CRAMP	CRAMP	GLLRKGGEKIGEKLKKIGQKIKNFFQKLVPQPEQ	[26,27]

CAMP/Camp, cathelicidin antimicrobial peptide; hCAP-18, human cationic antimicrobial peptide-18; LL-37, leucine leucine-37; CRAMP, cathelicidin-related antimicrobial peptide.

Beyond their antimicrobial functions, these cathelicidin peptides are also reported to have important immunomodulatory roles [30–35]. In humans, LL-37 has been associated with psoriasis, systemic lupus erythematosus, arthritis and atherosclerosis, whereas studies in murine models have shown that CRAMP influences disease processes such as atherosclerosis and autoimmune diabetes [36–42]. Bhusal and colleagues recently showed that murine CRAMP facilitates communication between astrocytes and microglia in experimental autoimmune encephalomyelitis (EAE), highlighting a potential parallel role for human LL-37 in multiple sclerosis (MS) [43]. CRAMP, largely produced by astrocytes, enhances IFN-γ-induced STAT3 signalling in microglia *via* formyl peptide receptors [43]. Consistent with this, mice lacking *Camp* gene showed lower rates of EAE and less IFN-γ production from T cells [13]. These findings provide insight into the previously unidentified function of CRAMP in mediating interactions between astrocytes and microglia. Another study found that human antimicrobial peptide LL-37 promotes chloride intracellular channel protein 1 (CLIC1) activation in mouse microglia, triggering neuroinflammation, excitotoxicity and Alzheimer’s disease-related neuropathological changes like amyloid and tau pathology, neurodegeneration and cognitive impairment [12]. Targeting the LL-37-CLIC1 axis may represent a potential therapeutic strategy for AD.

CRAMP may exert opposing role depending on its cellular source [44]. It was found that CNS-infiltrating neutrophils generate neutrophil extracellular traps (NETs) enriched with CRAMP, which are essential for the initiation of EAE. NET-associated CRAMP activates mouse dendritic cells *via* the cGAS/STING pathway, inducing IL-6 production and promoting a pathogenic Th17 response. In contrast, at later stages of the disease, CRAMP expression in mouse neurons plays a protective role by mitigating EAE severity. Knockdown of *Camp* in neurons exacerbates disease progression, whereas local administration of CRAMP at the peak of EAE facilitates disease remission [44]. These discrepancies from previous studies highlights the dual and context-dependent nature of CRAMP’s functions in neuroinflammation. The balance between its pro-inflammatory and anti-inflammatory effects is influenced by factors such as cellular source, extracellular signals, quantity and activation of specific signalling pathways. Bhusal et al. demonstrated the anti-inflammatory effects of CRAMP in the inflamed brain. Their study revealed that LPS-induced neuroinflammation led to increased CRAMP expression in various cell types such as mouse microglia, astrocytes and neurons [11]. CRAMP peptide treatment was shown to attenuate the pro-inflammatory effects of LPS in cultured microglial cells and mice. They also demonstrated that the capacity of CRAMP to decrease the LPS response in microglia was independent of the order in which microglia were exposed to CRAMP peptide and LPS^11^. A recent study found that *Cramp* knockout mice had greater infarct volumes, worse neurological outcomes, less endothelial cell proliferation and lower vascular density than wild-type mice at 7- and 14-days post-stroke [45]. CRAMP therapy was reported to increase angiogenesis-related gene expression in mouse brain endothelial cells after oxygen-glucose deprivation *in vitro*. Furthermore, blocking the CRAMP receptor CXCR2 reduced angiogenesis and neurological recovery after a stroke, but administering CRAMP peptide increased endothelial proliferation, angiogenesis and functional recovery. The work reveals that neutrophil-derived CRAMP plays a neuroprotective role in boosting angiogenesis and brain repair in the late stages of ischaemic stroke, making it a viable therapeutic target [45]. The dual, context-dependent nature of CRAMP’s effects on inflammation might be due to the complex and multifunctional nature of cathelicidin peptides in general. The specific mechanisms governing the pro-inflammatory versus anti-inflammatory activities of CRAMP appear to be influenced by multiple factors like the cellular environment, extracellular signals and the exact signalling pathways and transcription factors involved.

## Peptidoglycan recognition protein 1

Apart from their direct action against invading pathogens, some AMPs also function as pattern recognition receptors (PRRs). Peptidoglycan (PGN) is the major component of bacterial cell walls and one of the main microbial products recognized by the innate immune system. Moreover, it has been found that PGNs are ubiquitously present in the circulation and can cross the blood-brain barrier [46]. PGN recognition is mediated by several families of pattern recognition molecules, including peptidoglycan recognition proteins (PGRPs) [3]. Mammals have a family of four PGRPs, which were initially named PGRP-S, PGRP-L and PGRP-Iα and PGRP-Iβ (for ‘short,’ ‘long,’ or ‘intermediate’ transcripts, respectively). Subsequently, the Human Genome Organization Gene Nomenclature Committee changed their symbols to PGLYRP-1, PGLYRP-2, PGLYRP-3 and PGLYRP-4, respectively. This terminology is also used for mouse PGRPs [47].

PGRPs have recently emerged as potential key regulators of normal brain development and behaviour. A study found that *Pglyrp2* knockout mice have altered expression of the genes that are essential for brain circuit development and modulation [46,48]. Another study showed that *Pglyrp2* deficiency leads to major sex-dependent alterations in motor and anxiety-like behaviour, demonstrating that the effect of PGLYRP2 in the brain is modified by age, gender and neural circuitry [48,49]. PGLYRP1, also known as tumour antigen 7 (tag7) protein or PGRP-S, is another member of the mammalian PGRP family and is highly expressed in immune cells such as neutrophils, macrophages and eosinophils [50] and has been linked to several inflammatory conditions like rheumatoid arthritis [51], airway inflammation [50], coronary artery disease [52–54], asthma [55,56], oral inflammation [57–60] and cancer [61]. PGLYRP1 treatment to different cell types causes an increased expression of genes encoding pro-inflammatory cytokines [62,63]. Besides, *Pglyrp1* knockout mice are associated with a decreased inflammatory response when compared to wild-type animals [64]. *Pglyrp1* mRNA is expressed in the brain, with its level relatively increased with ageing [65], under sleep deprivation [66] and under ischaemic conditions [67].

**Figure 2.**
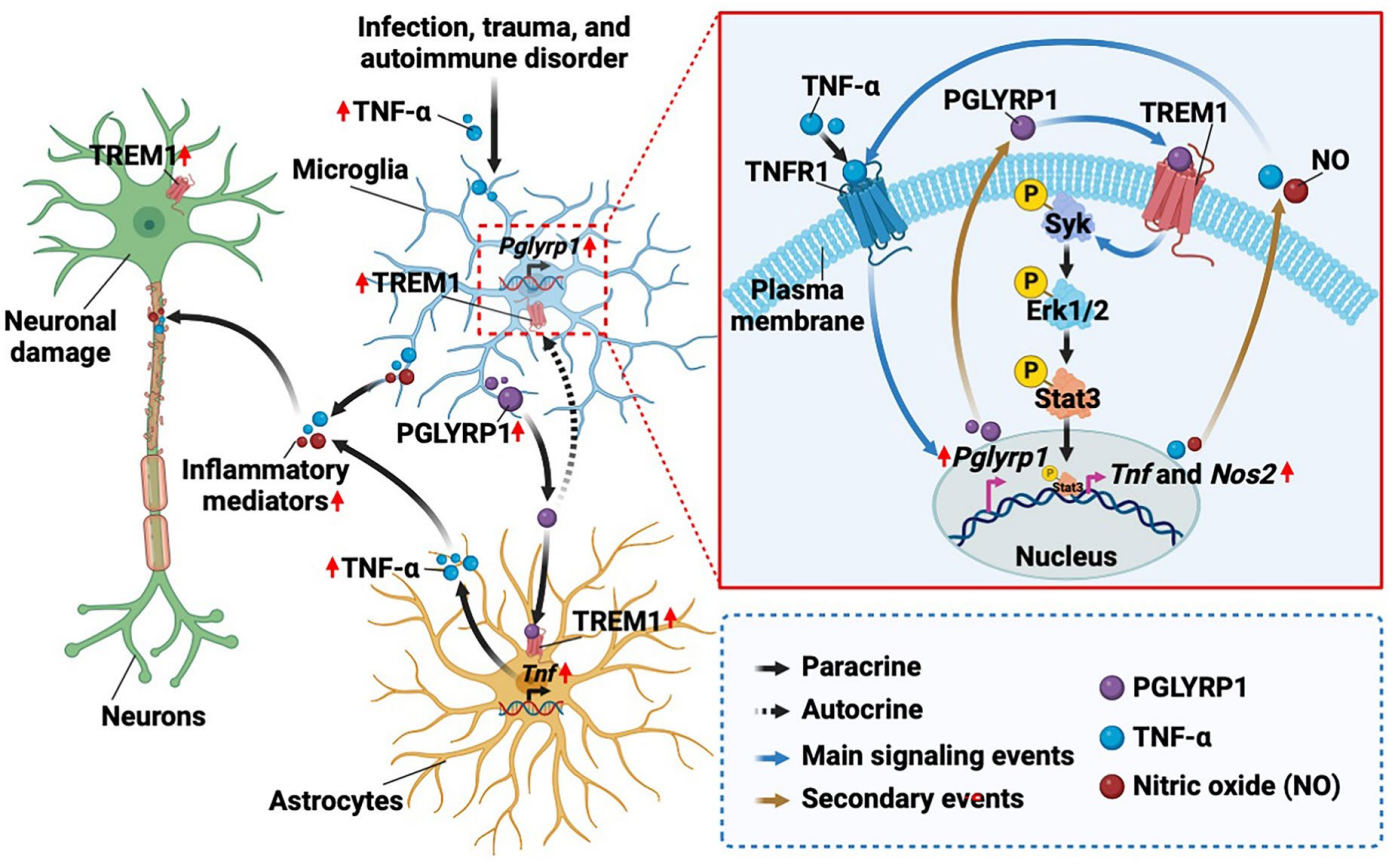
Role of microglial PGLYRP1 in neuroinflammation. Under inflammatory conditions induced by infection, trauma, or autoimmune diseases, microglia become activated and secrete the PGLYRP1 protein. This released PGLYRP1 acts on nearby cells, such as astrocytes, and on microglia themselves through TREM1 receptors. Ligation of PGLYRP1 to TREM1 triggers the activation of Syk-Erk1/2-Stat3 pathway in target cells, leading to their inflammatory activation and the expression/release of inflammatory cytokines like TNF-α. TNF-α, in turn, stimulates further PGLYRP1 expression in microglia, creating a positive feedback loop that exacerbates neuroinflammation. Blue arrows indicate main signalling events, and brown arrows denote secondary events. Abbreviations: Erk - Extracellular signal-regulated kinase; NO - Nitric oxide; Nos - Nitric oxide synthase; PGLYRP1 - Peptidoglycan recognition protein 1; Stat - Signal transducer and activator of transcription; Syk - Spleen tyrosine kinase; TREM - Triggering receptor expressed on myeloid cells; TNF - Tumor necrosis factor; TNFR - Tumor necrosis factor receptor. Created with BioRender. Adapted from Bhusal et al. (2024) [69].

The role of PGLYRP1 in neuroinflammation has not been investigated until recently. Genetic deletion of *Pglyrp1* protected against the development of EAE, which is a rodent model of MS, an autoimmune disease in the CNS [68]. *Pglyrp1*-deficient myeloid cells showed abnormalities in antigen presentation and T-cell activation, indicating that PGLYRP1 acts as a pro-inflammatory molecule in myeloid cells during autoimmunity. Targeting PGLYRP1 reduces autoimmune neuroinflammation by affecting myeloid cells’ pro-inflammatory activities and consequent T-cell activation in the CNS [68]. In line with these findings, Bhusal et al. found that PGLYRP1 is upregulated and mainly expressed by microglia during neuroinflammation [69]. They showed that PGLYRP1 and TNF-α mutually regulate each other’s expression, thereby amplifying neuroinflammation **(**Figure 2). PGLYRP1 enhances neuroinflammation through the TREM1-Syk-Erk1/2-Stat3 signalling pathway, pointing to a potential therapeutic target for neuroinflammatory diseases [69]. While microglia appear to be the primary source, astrocytic PGLYRP1 may also cause neuroinflammation and gliosis. Further studies using cell-specific deletion models and co-culture techniques could shed light on the precise functional role of astrocytic PGLYRP1. Further, most of the research has centred on PGLYRP1’s function as a ligand for different receptors. Recent discoveries reveal that PGLYRP1 can also serve as an intracellular receptor for the disaccharide motif of GMTriP-K (lysine N-acetylglucosamine N-acetylmuramic tripeptide, a peptidoglycan-derived disaccharide fragment generated *via* lysozyme digestion of bacterial cell walls), modulating transcriptional responses in macrophages that play a role in controlling intestinal inflammation [70]. This novel function warrants further exploration, particularly in the context of neuroinflammatory conditions. There are concerns regarding the potential impact of targeting PGLYRP1 on its antibacterial function and host vulnerability to bacterial infections. PGLYRP1 is recognized to participate in innate immune responses against microorganisms. Strategies for modifying PGLYRP1 in neuroinflammatory disorders must be carefully developed to maintain its antimicrobial action while reducing its pro-inflammatory effects in the nervous system. Some possible ways may include: i) developing CNS-specific inhibitors or modulators of PGLYRP1 that do not impact its peripheral antibacterial action, ii) targeting downstream signalling pathways associated with PGLYRP1-mediated neuroinflammation, or iii) exploring how PGLYRP1 expression and activity vary by cell or tissue type to control its effects in the CNS.

## Lipocalin-2

Lipocalin-2 (LCN2), also known as neutrophil gelatinase-associated lipocalin (NGAL), plays a critical role in the body’s innate immune response by inhibiting bacterial growth [71,72]. LCN2 expression is systemically induced following infection with pathogens like *Escherichia coli* [73]. *Lcn2* knockout mice are more susceptible to bacterial infections, with increased mortality rates due to the lack of LCN2’s bacteriostatic activity and decreased immunological function [74]. LCN2 restricts bacterial growth and modifies immune cell function, playing a crucial role in maintaining gut homeostasis and preventing dysbiosis and inflammation [75]. Mechanistically, LCN2 attaches to and traps bacterial siderophores, tiny molecules that bacteria employ to extract iron from their host. By sequestering these siderophores, LCN2 deprives bacteria of iron essential for their growth and reproduction [76].

Neurodegenerative diseases exhibit abnormalities in CNS iron metabolism, consistently presenting with iron overload [77]. This observation has spurred researchers to investigate the involvement of LCN2 in neuroinflammation associated with these disorders. In a healthy, normal state, the brain exhibits very low or negligible levels of LCN2. However, this expression pattern changes dramatically when the brain experiences injury or inflammation. The earliest evidence of LCN2 overexpression in the brain came from peripheral turpentine-induced inflammation [78]. Furthermore, the discovery that LCN2 is expressed in CNS astrocytes and microglia [79,80] marked a turning point in the investigation of LCN2 activity in a variety of neuroinflammatory disorders (reviewed in refs. [71,81–83]. Recently, researchers have focused on the role of LCN2 in ischaemic and traumatic brain injury conditions, reporting elevated *Lcn2* mRNA/protein levels in mouse models of TBI and ischaemia, as well as increased LCN2 expression in human patients with chronic traumatic encephalopathy [84]. In a study by Kim et al. they found that LCN2 mediates secondary damage following traumatic and ischaemic brain injury in mice by promoting neuroinflammation and suppressing the expression of neurotrophic factors [84]. In line with this, a study by Li et al. demonstrated that the LCN2/24p3R interaction mediates astrocyte pyroptosis *via* NLRP3 inflammasome activation following cerebral ischemia/reperfusion injury [85]. More recent findings showed a strong positive correlation between LCN2 and IL-6 in acute traumatic brain injury (TBI) patients [86]. Furthermore, *in vitro* studies using primary mouse glial cultures demonstrated a reciprocal regulatory loop: IL-6 enhanced astrocytic LCN2 expression *via* STAT3 signalling, while LCN2, in turn, upregulated microglial IL-6 production through the NF-κB pathway [86]. Further, the *Lcn2* deficiency reduces gliosis, pro-inflammatory cytokines and disease severity in mouse models of Krabbe disease [87] and acute glaucoma [88]. Overall, these insights suggest that targeting LCN2 could offer a promising therapeutic strategy for mitigating neuroinflammation in a range of neurological disorders.

Aside from brain injuries, an important finding has highlighted the association of high LCN2 levels in patient blood and brain metastases with disease progression and poor survival across multiple cancer types [89]. Overall, LCN2 plays a pivotal role in initiating and sustaining glial cell activation, as well as regulating their crosstalk. More importantly, its level could be estimated in the plasma and cerebrospinal fluid (CSF) during neuroinflammatory conditions. Thus, LCN2 exhibits considerable promise as a biomarker, suggesting that it may be useful in diagnostics and prognostics. Beyond its use as a biomarker, LCN2 may also serve as a target for therapeutic interventions. Even though LCN2 has been extensively studied in a broad range of neurological disorders, the exact mechanisms underlying different functions of LCN2 are still not fully understood, which emphasizes the need for more research in this field.

## Molecular mechanisms underlying AMP modulation of neuroinflammation

Antimicrobial peptides (AMPs) are categorized based on their structure and mode of action [3]. Among these, cationic peptides, such as cathelicidins, display potent antibacterial activity by targeting negatively charged bacterial membranes [1]. Clinically, LL-37 levels rise in the CSF during bacterial meningitis, and the CSF shows antimicrobial activity, suggesting AMPs contribute to pathogen elimination in the nervous system [9,89]. Furthermore, cationic antimicrobial peptides have been demonstrated to bind and sequester free bacterial LPS and lipoteichoic acids (LTAs), thereby inhibiting the activation of Toll-like receptors, such as TLR2 and TLR4 [90,91]. This mechanism may underlie their potential anti-inflammatory effects within the nervous system [11]. In addition to these antimicrobial and anti-inflammatory roles, LL-37 also acts as a chemoattractant, recruiting various leukocytes and CD4^+^ T cells through the FPRL1 receptor and mast cells *via* the MrgX2 receptor. However, whether these chemoattractant properties are retained in the context of neuroinflammation remains an open question [92]. In the nervous system, CRAMP/LL37 are expressed by neurons and glial cells. Upon release, these peptides stimulate glial cells to produce pro-inflammatory cytokines and chemokines, which in turn may affect neuronal health. The dual role of these peptides as both ‘pro-inflammatory’ and ‘anti-inflammatory’ agents highlights their crucial function in maintaining immunological balance within the nervous system. This duality underscores the importance of context and experimental design in interpreting previous research.

Defensins, another class of small cationic AMPs, are divided into two main groups in humans: α-defensins and β-defensins, both of which exhibit broad-spectrum antimicrobial activity. Emerging evidence suggests that β-defensins may play a role in neuroimmune function and neurodegeneration [93]. Notably, human β-defensin-2 (hBD-2) levels are significantly elevated in the serum and CSF of patients with AD compared to age-matched controls [94]. In addition, human β-defensin-1 (hBD-1) is found within hippocampal astrocytes, neurons and the choroid plexus, and its expression is increased in patients with AD [95]. Interestingly, hBD-2 and hBD-3 are not similarly localized or upregulated in these regions. While these peptides are observed in the CNS during inflammatory conditions, their precise roles in neuroinflammation and disease pathogenesis remain speculative and largely correlational. Comprehensive studies to elucidate their exact mechanisms of action are currently lacking, highlighting a critical gap in our understanding of their contributions to neuroimmune regulation and neurodegenerative diseases.

Unlike cathelicidins and defensins, PGLYRPs, such as PGLYRP1, PGLYRP3 and PGLYRP4, kill bacteria by interacting with bacterial cell wall peptidoglycan rather than by permeabilizing membranes. Binding of PGLYRPs to peptidoglycan induces bacterial oxidative stress, leading to bacterial death, while also modulating inflammatory responses. Among these proteins, PGLYRP2 is unique due to its N-acetylmuramoyl-L-alanine amidase activity, which enzymatically degrades peptidoglycan into smaller fragments. This enzymatic activity contributes to immune regulation and enhances bacterial clearance. As discussed above, recent research highlights the critical role of PGLYRP1 in mediating neuroinflammation. Additionally, PGLYRP1 can form a stable complex with heat shock protein 70 (Hsp70), called the Tag7-Hsp70 complex, which exhibits cytotoxic effects on tumour cells by inducing programmed cell death pathways, including apoptosis and necroptosis, *via* TNFR1 receptor signalling [96]. These HSPs are integral components of the nuclear factor erythroid 2-related factor 2 (Nrf2)-mediated cellular defense network, where they synergize with vitagenes to maintain redox homeostasis and enhance cytoprotection [97,98]. Given that HSPs play a crucial regulatory role in maintaining cellular redox homeostasis—an activity that is markedly upregulated in MS brains [99]—it is plausible that such complexes between AMPs and HSPs may potentially exacerbate oxidative stress, thereby contributing to the severity of disease. As our understanding of PGLYRPs continues to grow, more detailed studies are needed to elucidate their complex roles and evaluate their potential as therapeutic targets for neurological disorders.

Some other antimicrobial proteins such as LCN2, play a critical role in host defense by sequestering bacterial siderophores, thereby limiting iron availability to pathogens. Beyond its antimicrobial functions, LCN2 interacts with its cell membrane receptor (24p3R), triggering signalling pathways, such as NF-κB phosphorylation and the JAK-STAT pathway, influencing glial cell activation and regulating cytokine production and release. Additionally, LCN2 has been implicated in cellular apoptosis, oxidative stress and blood-brain barrier disruption [100]. Similarly, S100 proteins exhibit potent antimicrobial activity through a mechanism known as ‘nutritional immunity.’ By sequestering essential metal ions such as zinc and manganese, S100 proteins deprive pathogens of nutrients necessary for their growth and survival [101]. In the context of neuroinflammation, S100 proteins can act as damage-associated molecular patterns (DAMPs), activating pattern recognition receptors such as TLRs and the receptor for advanced glycation end products (RAGE) [102,103]. This activation initiates inflammatory cascades, recruiting immune cells to sites of infection or injury in the CNS. Additionally, S100 proteins modulate the function of glial cells, particularly microglia and astrocytes, by influencing their activation states and promoting the release of pro-inflammatory mediators [104]. These activities position both LCN2 and S100 proteins as key players in the regulation of neuroinflammatory responses.

## Perspective and conclusion

AMPs are an evolutionarily conserved defense mechanism. Evidence from both murine models and human studies demonstrates that AMPs can either exacerbate or mitigate neuroinflammation depending on cellular source and disease stage. These findings underscore that AMPs are not only antimicrobial agents but also context-dependent modulators of immune signalling, glial activation and neuronal survival. By elucidating the expression and the mechanisms by which AMPs influence neuroinflammation, researchers can identify novel biomarkers or therapeutic targets for the early detection or treatment of neuroinflammatory and neurodegenerative diseases with significant translational potential.

However, several challenges remain in the clinical development of AMPs. Rapid degradation by proteases in biological fluids reduces their bioavailability and efficacy *in vivo*. Additionally, some AMPs exhibit cytotoxicity towards mammalian cells at therapeutic concentrations, limiting their clinical use. Further, the cationic and amphipathic nature of AMPs results in poor bioavailability and difficulty crossing physiological barriers like cell membranes and the blood-brain barrier. As proteins, AMPs may also elicit undesirable immune responses upon systemic administration, limiting repeated dosing. Furthermore, cost-effective large-scale production of AMPs remains an obstacle to commercialization. Despite the currently limited clinical use of AMPs, recent advancements in the field have addressed significant challenges, paving the way for a significant increase in the therapeutic application of synthetic AMPs. Innovations in multidimensional, computer-assisted peptide design, combinatorial optimization and *in silico* modelling are expected to accelerate the discovery of next-generation therapeutic peptides. These peptides will likely offer broader coverage and more precise anti-infective and immunomodulatory properties.

## Data Availability

Data sharing is not applicable to this article as no new data were created or analyzed in this study.
